# SARS-CoV-2 and EBV; the cost of a second mitochondrial “whammy”?

**DOI:** 10.1186/s12979-021-00252-x

**Published:** 2021-10-30

**Authors:** Alistair V.W. Nunn, Geoffrey W. Guy, Stanley W. Botchway, Jimmy D. Bell

**Affiliations:** 1 Research Centre for Optimal Health, Department of Life Sciences, University of Westminster, W1W 6UW London, UK; 2 The Guy Foundation, Dorset, UK; 3 Department of Biological and Medical Sciences, UKRI, STFC, Central Laser Facility, Oxford Brookes University, OX1 10QX Oxford, UK

**Keywords:** SARS-CoV-2, Epstein Barr Virus, Mitochondria, Long COVID, Ageing, Cost, Inflammaging, Immunosenescence

## Abstract

We, and others, have suggested that as the SARS-CoV-2 virus may modulate mitochondrial function, good mitochondrial reserve and health could be key in determining disease severity when exposed to this virus, as the immune system itself is dependent on this organelle’s function. With the recent publication of a paper showing that long COVID could be associated with the reactivation of the Epstein Barr Virus, which is well known to manipulate mitochondria, we suggest that this could represent a second mitochondrial “whammy” that might support the mitochondrial hypothesis underlying COVID-19 severity and potentially, the occurrence of longer-term symptoms. As mitochondrial function declines with age, this could be an important factor in why older populations are more susceptible. Key factors which ensure optimal mitochondrial health are generally those that ensure healthy ageing, such as a good lifestyle with plenty of physical activity. The ability of viruses to manipulate mitochondrial function is well described, and it is now also thought that for evolutionary reasons, they also manipulate the ageing process. Given that slowing the ageing process could well be linked to better economic outcomes, the link between mitochondrial health, economics, COVID-19 and other viruses, as well as lifestyle, needs to be considered.

## Background

As mitochondria are key in the immune response [1], we have previously suggested that as SARS-CoV-2 may modulate mitochondrial function it may have a greater adverse effect in those with poorer mitochondrial health, such as the elderly and those displaying evidence of the metabolic syndrome [2]. We are not alone in this thinking [3–5], which is why it has been suggested that high aerobic fitness could be beneficial [6]. In fact, data continue to support a role for mitochondrial dysfunction and metabolic perturbation in the pathogenesis of this condition [7, 8] – including coincident hyperglycaemia that seems to be associated with more severe disease [9]. In short, good mitochondrial health and reserve could well be important components in the resistance to SARS-CoV-2. Although there is building evidence for this [10], the idea is still not yet mainstream.

However, it now seems that “long COVID” could also be related to reactivation of the Epstein Barr Virus (EBV), which lies dormant in a very high percentage of the population [11]. For instance, there is a correlation with impaired lymphocyte subpopulation count, EBV load and severity of disease [12], as well as evidence that EBV can induce angiotensin-converting enzyme 2 (ACE2) expression and SARS-CoV-2 entry to epithelial cells [13]. Critically, given that more than half of survivors of COVID-19 appear to have long term symptoms (post-acute sequelae of COVID-19 [PASC]) or “long-COVID” [14], the described association of EBV with mitochondrial dysfunction [15] could play a key role in susceptibility and recovery from SARS-CoV-2. Furthermore, the recent discovery that mitochondrial function is essential for sustained killing by cytotoxic T cells, for instance, of virally infected cells [16], is perhaps also suggestive, as EBV can also infect T cells [17]. In this regard, it is perhaps relevant that in people with SARS-COV-2 a subset of T cells with an altered metabolic profile, which are prone to mitochondrial apoptosis and resultant lymphopenia,  as well as enhanced numbers of myeloid-derived suppressor cells, are associated with disease severity and altered voltage dependent anion channel 1 (VDAC1) expression – a pivotal mitochondrial protein [18].

In effect, EBV could tip an already compromised system into a self-perpetuating metabolic-oxidative stress-inflammatory spiral that could underly many of the symptoms of long COVID. The likelihood of this happening could be increased in people who already have sub-optimal mitochondrial function associated with a sedentary lifestyle. In this regard, a genetic and phenotypic analysis of the causal relationship between ageing and COVID-19 seems to indicate that accelerated biological ageing is a key factor in susceptibility risk, with both altered Notch signalling and B cell functioning being important [19].

### EBV modulates mitochondria – a second whammy?

The association between EBV-induced chronic fatigue syndrome with mitochondrial dysfunction was suggested many years ago [15]. EBV can alter mitochondrial dynamics and DNA replication [20, 21], as well as interact with latent membrane protein 1 (LMP1) and dynamin-related protein 1 (Drp1), enhancing glycolysis [22]. It can also induce metabolic reprogramming in monocytes, which is associated with decreased autophagy and mitochondrial biogenesis. This enhances apoptosis and reduces immune surveillance [23]. EBV also modulates c-Myc activity and glutaminolysis and thus aerobic glycolysis and enhances the activity of glutaminase-1 (GLS-1) isoforms in mitochondria, which explains its association with cancer [24]. This therefore raises the possibility that reactivation of the EBV serves as a second mitochondrial “whammy”, leading to a sustained inflammatory response and the classical symptoms of brain fog, tiredness and weakness. This has many similarities to cytokine induced sickness behaviour associated with other conditions, such as cancer, cancer treatment, multiple sclerosis, as well as virally-induced fatigue syndrome, where mitochondrial dysfunction could also play a role [25]. The key here is that dysfunctional mitochondria can be a source of oxidative stress, which in turn, further compromises mitochondrial recovery due to feedback and their role in inflammatory signalling [26]. It is thus possible that even if the cause of the first whammy, the corona virus, is cleared, this imbalance can continue for some time; the second EBV whammy, whether coincident, or caused by reactivation due to reduced immune function, would only make this worse.

## Mitochondria are more than just “powerhouses” of the cell

At the most basic level the mitochondrion must balance metabolic priorities between energy production, growth, defence and the management of oxidative stress. The key to understanding this is that mitochondria are not just “powerhouses”, but also sense the cellular environment, control growth, inflammation, senescence and death. Having a good mitochondrial reserve enables the cell to be flexible and multi-task, whether it is in a neuron or an immune cell; loss of this flexibility certainly plays a role in ageing-related immunosenescence and the concept of “inflammaging” [27]. This is why physical activity is generally viewed as being a very good medicine, as all mitochondria in the body communicate with each other via myokines; the stress of exercise doesn’t just induce an adaption in muscle, but in all other bodily systems and improves overall robustness [28]. This perhaps reflects our evolutionary heritage where movement was normal and became canalised as a necessary factor to maintain fitness in a calorie restricted environment; not moving and eating too much thus takes humans outside our evolutionary “flight envelope” [29].

## Long COVID is not just a sequalae of the very ill

It may well be relevant that pathogens such as viruses appear to manipulate the ageing process for their own evolutionary benefits, which could be viewed as an extension of the disposable soma theory of ageing [30]. This might suggest that even in people who do not exhibit particularly severe symptoms with the initial SARS-CoV-2 infection, reactivation of EBV, or other viruses, could trigger a secondary longer-term syndrome if their mitochondrial reserve is low. In terms of prevention and treatment, not only would this continue to support physical activity as being key, but also diets high in plant defence compounds with pleiotropic actions that are known to modulate mitochondrial function, induce resolution of inflammation, as well as displaying anti-pathogen function. This concept could also extend to existing drugs that also have pleiotropic actions involving modulation of mitochondrial function, such as metformin or steroids. It might also hint that mild calorie restriction, which can enhance mitochondrial function and reduce inflammation, could also play a role [2]. It has also been suggested a ketogenic diet might help, as mitochondria are key in the metabolic adaptation [31].

Viral reactivation might also indicate why there is some variability in response to treatment, as it is possible that each patient could have a mix of reactivated viruses, or other pathogens that complicate interpretation of efficacy as their bodies try to balance the multiple functions of mitochondria. A case in point is the potential for gut microbiota to affect how apparently healthy subjects respond to COVID-19 [32] and that there is cross-talk between gut microbiota and mitochondria, for instance, after exercise [33]. In short, having a healthy mitochondrial system could prevent a “domino” effect leading to a tipping point into a chronic inflammatory state, and if already infected, mitochondrial support and protection may provide a means to break the cycle. It therefore continues to suggest that not getting the virus in the first place, or being vaccinated, are key strategies. Indeed, a study in Switzerland suggested that 34.8 % of participants in an outpatient setting had post-acute symptoms seven months after diagnosis of COVID-19 [34].

## Conclusions – the cost of mitochondrial ill-health

As is fairly well established, a poor lifestyle accelerates the ageing process leading to morbidity expansion [29], which reduces the capacity of an individual to work and increases the load on society [35]. Add to this that viruses may also influence the ageing process [30], then a poor lifestyle coupled with increased viral load is going to further accelerate ageing across society and negatively affect the economy. Indeed a recent analysis suggests that slowing ageing across society could be, by far, the most sensible thing to do economically [36]. Given the well described link between mitochondrial function and ageing, especially of the immune system [37], then it could be argued there is also one between economics and mitochondrial health. Hence, the paper by Gold et al.[11] hints at another very important angle that governments, and healthcare providers, may need to consider: overloading ill-prepared mitochondria could cost money – and this overload could come in the guise of other viruses.

## Data Availability

not applicable
